# Investigating the Validity Evidence of the Swedish TriPM in High Security Prisoners Using the PCL-R and NEO-FFI

**DOI:** 10.3389/fpsyt.2021.704516

**Published:** 2021-11-19

**Authors:** Malin Pauli, Hannibal Ölund Alonso, Jenny Liljeberg, Petter Gustavsson, Katarina Howner

**Affiliations:** ^1^Department of Clinical Neuroscience, Karolinska Institutet, Stockholm, Sweden; ^2^National Board of Forensic Medicine, Stockholm, Sweden; ^3^Forensic Psychiatric Care Stockholm, Stockholm Healthcare Services, Stockholm, Sweden

**Keywords:** psychopathy, triarchic psychopathy model, triarchic psychopathy measure, prison inmates, construct validity

## Abstract

The triarchic model of psychopathy was developed to bridge opposing descriptions of psychopathy by separating the core construct in three domains; boldness, meanness, and disinhibition. The Triarchic Psychopathy Measure (TriPM) operationalizes the model through a 58-item self-report questionnaire. The current study examined the psychometric properties of the Swedish translation by investigating intercorrelations and associations to expert-rated psychopathy, general personality and psychopathy-related traits in male high-security prisoners (*n* = 191). Psychopathy rated with TriPM and the Psychopathy Checklist Revised (PCL-R) demonstrated expected convergence, as did empathy and impulsivity measures. The Disinhibition and Meanness scales were highly correlated, indicating that the scales might not be adequately differentiated. Nevertheless, the divergent association patterns to other important variables, particularly neuroticism and empathy, also points to meaningful differences. However, the lack of association between Disinhibition and Boldness may put into question if these domains are related at all, especially since there was a lack of similarity in the association patterns with other clinical variables. The influence of antisocial behavior in the TriPM operationalization might amplify the similarities of the Meanness and Disinhibition scales, while diluting the associations between Meanness and Boldness. In conclusion, the Swedish TriPM is effective in measuring the domains of triarchic model in forensic settings, even though a revision of the scales might improve the psychometric properties of the instrument.

## Introduction

Psychopathy is a pervasive and maladaptive pattern of personality traits, including reduced empathy, callousness, lack of remorse, grandiosity, and interpersonal dominance (1). The disorder has mainly been studied and assessed in forensic settings, as it is overrepresented among offenders and of clear interest for risk management within correctional services. This does, however, place a large focus on criminal and antisocial behavior rather than the intrinsic personality traits of the disorder, limiting the tools used for evaluating psychopathy in non-forensic populations. There has been a long, ongoing scientific debate of what the disorder encompasses, with two main questions; the first being if psychopathy is a unitary entity or a combination of interconnected, yet distinguishable traits, and the other if criminality and antisocial behavior should be regarded as a core symptom or a result of the disorder (2). This reflects the heterogeneity of the construct and the difficulties in defining what is the core traits vs. the consequences of psychopathy.

The triarchic model of psychopathy, introduced by Patrick et al. (3), is an integrative model striving to bridge competing current and historical descriptions of psychopathy. It is based on three phenotypic constructs or domains; boldness, meanness, and disinhibition, which can be understood and measured separately. Boldness encompasses social dominance, emotional resilience, and venturesomeness. Meanness is defined as an aggressive competitiveness without regard for others. Disinhibition refers to a general disposition of problems with impulse control and emotional regulation. According to the model there are two underlying factors contributing to the development of the respective domains. Patrick et al. (3) hypothesize that disinhibition and meanness stem from difficult temperament and therefore are moderately interrelated. Meanness and boldness are theorized to be somewhat interrelated, due to the etiological contribution of low dispositional fear, while disinhibition and boldness are thought to be minimally interrelated. Since the triarchic model was presented, literature supporting its conceptual framework and validity is rapidly growing (4).

Boldness, also referred to as fearless dominance in other conceptualizations of psychopathy, is associated with low levels of anxiety and depression, as well as positive traits such as coping and well-being (5–8). It is described as a semi-adaptive trait, that might serve as a protective factor against the consequences and manifestations of meanness and disinhibition (9). The importance of boldness in psychopathy is controversial, with some authors stating that it is central for identifying adaptive features such as social assertiveness and emotional resilience, representing an important dividing line distinguishing psychopathy from antisocial personality disorder. Others conclude that boldness is neither necessary nor sufficient for psychopathy, but might be thought of as a diagnostic specifier [e.g., (10–12) for a thorough discussion]. A recent meta-analysis by Sleep et al. (13), covering the relationship between the triarchic model and alternative measures of psychopathy, demonstrated that boldness was not associated to constructs that are theoretically relevant of psychopathy (e.g., antagonism). Furthermore, the authors discuss that the evidence of boldness as related to adaptive functioning is difficult to reconcile with the view that psychopathy is principally a highly impairing personality disorder.

Most studies to date use the Triarchic Psychopathy Measure (TriPM) (14) to operationalize the triarchic model. The TriPM, a 58-item self-report questionnaire, was developed to encompass the triarchic domains and the items are divided into the three subscales of the model. Of these, the Meanness (19 items) and Disinhibition (20 items) scales were drawn from the 415 items of the Externalizing Spectrum Inventory (ESI) (15), which was developed to assess disinhibition and externalizing psychopathology (i.e., substance abuse, impulsivity, aggressiveness, conduct disorder, and antisocial behavior). The Boldness scale (19 items) is a brief version of the 130 item Boldness Inventory (16), developed as a refined measurement of the fearless dominance construct encompassing fearlessness in social interactions, resilience to emotional stress, and a daring disposition. The triarchic model primarily conceptualizes psychopathy as separable, yet interrelated domains rather than emphasizing the disorder as a global unitary construct. However, there is a broad consensus that psychopathy is a cohesive phenomenon, albeit multidimensional. In a meta-analysis by Sleep et al. (13) of 84 studies using various instruments used to assess the triarchic domains of psychopathy, the authors found that boldness showed a small positive association to meanness (*r* = 0.16) and a negative association with disinhibition (*r* = −0.05), whereas meanness and disinhibition were positively intercorrelated (*r* = 0.53).

Most psychometric studies of the TriPM have been performed in non-forensic populations, meaning that they have not been able to make use of the Psychopathy Checklist Revised (PCL-R) (17), which is currently the most extensively validated method for measuring psychopathy in forensic settings. The PCL-R model characterizes psychopathy as personality traits linked to deviant patterns of interpersonal (facet 1) and affective function (facet 2), as well as by behavioral deviance linked to an impulsive lifestyle (facet 3) and antisocial behavior (facet 4). There are to date few studies that have investigated the validity of the TriPM in regards to the PCL, and all have been exclusively in male populations. A study of 152 prison inmates performed by Wall et al. (18) found that both TriPM Meanness and Disinhibition were associated to PCL-R total score (*r* = 0.35 and 0.31, respectively), Facet 3 (*r* = 0.31 and 0.38) and Facet 4 (*r* = 0.33 and 0.44). The Meanness subscale was also associated to Facet 2 (*r* = 0.31). Boldness scores were however only associated to Facet 1 (*r* = 0.29). Utilizing the PCL Screening version (PCL:SV) (19) in a sample of 99 offenders in a custodial institution (20) likewise found that only Meanness and Disinhibition scales were associated with the total PCL:SV score (*r* = 0.27 and 0.21 respectively). In regards to PCL:SV facets, TriPM Meanness was associated to affective function (*r* = 0.35), Disinhibition with impulsive lifestyle (*r* = 0.28), and Boldness to interpersonal functioning (*r* = 0.21). Lastly, Yoon et al. (21) found that, in a population of 152 prison inmates, the Meanness and Disinhibition subscales of the TriPM were correlated to PCL-R Facet 2 (*r* = 0.26 and 0.26), Facet 3 (*r* = 0.45 and 0.63) and Facet 4 (*r* = 0.43 and 0.65). As opposed to previous studies, they did not find an association between the Boldness scale and Facet 1, nor with any other PCL-R facet.

As the PCL model is mainly of use in forensic settings, another strategy in defining psychopathy is by using dimensions of normal personality such as a five-factor model profile (FFM) (22). This is especially useful as it may function as a translation of the conceptual framework of different models of psychopathy and also providing a means to linking them to the broad empirical base of personality research (23). Previous research has demonstrated that the FFM dimensions of neuroticism, extraversion, openness, agreeableness and conscientiousness can be used to provide discriminant evidence for the triarchic model, with distinct patterns representing the respective domains (24–34). TriPM Boldness is chiefly linked to high Extraversion (*r* = 0.36 to 0.74) and low Neuroticism (*r* = −0.43 to −0.73), whereas Meanness and Disinhibition are both associated to low Agreeableness (*r* = −0.45 to −0.82 and −0.31 to −0.51, respectively) and Conscientiousness (*r* = −0.22 to −0.54 and −0.39 to −0.69, respectively). Furthermore, the Disinhibition scale is also associated to high Neuroticism (*r* = 0.18 to 0.51). Hyatt et al. (34) argue that the TriPM Boldness, Meanness, and Disinhibition scales might even be redundant to a FFM trait-based approach in describing psychopathy.

In addition to normal personality, there are several core traits that are of conceptual importance and intersect the construct of psychopathy, of which impulsivity and a lack of empathy are central. Previous studies have shown that self-reported impulsivity is mainly associated with TriPM Meanness and Disinhibition domains (9, 30, 35). However, other disorders characterized by reduced impulse control, such as attention-deficit hyperactivity disorder (ADHD), are also linked to delinquent behavior and criminality (36). In a previous report from the current sample we found that primarily TriPM Disinhibition, but also Meanness, were independently associated to self-reported ADHD symptoms (37). Nonetheless, as the overlap was quite salient, we concluded that the Disinhibition scale in particular was not adequately distinguishable from self-reported ADHD symptoms in offenders. In regards to empathy, TriPM Meanness is negatively associated with most aspects of the construct, while Boldness is negatively associated specifically to personal distress in tense social interactions (24, 38–41).

## Rationale

The current study aimed to examine the psychometric properties of the Swedish TriPM version by examining the association validity evidence in a Swedish high security prison sample. Specifically, based on the theoretical outline of the triarchic model and previous research of the measurement model, we investigated if the TriPM showed expected intercorrelations and associations using expert-rated psychopathy, self-rated normal personality traits, as well as the psychopathy-associated traits of empathy and impulsivity. The study focused on exploring the following hypotheses:

*Hypothesis 1*: The TriPM domains represent interrelated constructs. Meanness and Disinhibition are most strongly associated, while Boldness is mainly associated with Meanness but also, to a small degree, Disinhibition.

*Hypothesis 2*: Psychopathic traits assessed by TriPM are convergent with PCL-R based psychopathy, but the triarchic domains demonstrate specific patterns of associations with PCL-R total and facet scores. When controlling for domain co-variance, there is a positive association between Boldness and Facet 1 as well as Facet 2; Meanness and Facet 2; and Disinhibition to Facet 3 and 4.

*Hypothesis 3*: The TriPM domains are translatable in terms of general personality, as measured with NEO-FFI, empathy assessed by IRI and impulsivity measured through BIS-11. Boldness can be described in terms of high Extraversion and Openness, as well as low Neuroticism. Meanness is characterized mainly by low Agreeableness, Conscientiousness, and empathy. Disinhibition is characterized by high Neuroticism, in addition to low Conscientiousness and Agreeableness. Moreover, Disinhibition has a strong association to impulsivity.

## Materials and Methods

### Participants

The participants were male offenders recruited from all seven high security prisons in Sweden, as previously described in Pauli et al. (37). As the subjects were also part of a genetic study, all participants were of Swedish ethnicity. In total, we invited 309 inmates to participate, of which 206 agreed (67%). Due to missing or invalid data, 12 participants were excluded. To assess inattentive or careless responding, we calculated scores on the Triarchic Assessment Procedure for Inconsistent Responding (TAPIR) with a cut-off score of ≥17 in order to achieve specificity of <90% in an correctional setting (42). Three participants (1.5%) were identified as potential inconsistent responders of the TriPM and were excluded from data analysis, leaving a final study sample of 191^1^.

Participants were 22–65 years old with a mean age of 37.1 (SD 11.1). Approximately half of them (48%) had completed high school education and 42% reported having a history of childhood adversity. Most participants (80%) had a history of violence and 27% had committed lethal violence, while 17% had a history of sexual offending. Nearly half (49%) recounted having been diagnosed with antisocial personality disorder (ASPD), while a third (33%) stated a diagnosis of ADHD. The prevalence of self-reported substance abuse was 65%.

### Procedure

Participants were interviewed within the prisons by a clinically experienced research assistant using a semi-structured interview for PCL-R scoring (17) as well as a structured protocol regarding socioeconomic factors and demographic information. The participants correctional files were reviewed for collateral information. All participants were administered self-rating forms to assess triarchic psychopathy, normal personality, empathy, and impulsivity. Missing values were calculated by mean value computations (i.e., replacing missing values in each subscale with the mean value of the respective subscale to adjust the score). As compensation for their contribution, participants were awarded a telephone card for use within the prison services.

### Psychometric Instruments

#### Triarchic Psychopathy Measure

Participants completed the TriPM (14), which has previously been translated to Swedish (43). The self-report questionnaire contains 58 items, divided into the three triarchic domains. Items are statements about the participant (e.g., “I don't mind if someone I dislike gets hurt”) that are rated on a 4-point Likert-type scale with response options 0 (false), 1 (mostly false), 2 (mostly true), and 3 (true), though some items are scored in a reverse manner. This yields a maximum score of 176.

#### Psychopathy Checklist Revised

The PCL-R (17) is a 20-item rating scale assessing psychopathy through a semi-structured expert interview in addition to a collateral review of file information. The items are scored as absent (0), present to some degree (1), or fully present (2), with a maximum total score of 40.

#### NEO Five-Factor Inventory

Personality traits according to the five-factor model were assessed using the NEO-FFI (22). The self-report instrument consists of 60 items that assess the five personality dimensions; neuroticism, extraversion, openness, agreeableness, and conscientiousness, measured by 12 items, respectively. Items are rated on a 5-point scale, ranging from 0 (strongly disagree) to 4 (strongly agree). As it is a short-version of the NEO-PI-R (22), it does not allow for assessment of the personality domain sub-scale facets.

#### Barratt Impulsiveness Scale

Impulsivity was measured by using the 30-item self-report questionnaire BIS-11 (44). It consists of statements describing impulsive or non-impulsive (for reverse scored items) behaviors, that are scored between 1 (“rarely/never”) and 4 (“almost always/always”). This results in total scores ranging between 30 and 120, where a higher total score indicates a higher degree of impulsiveness.

#### Interpersonal Reactivity Index

The IRI (45) is a 28-item self-rating instrument assessing different aspects of empathy. The items consist of statements to be rated on a 5-point scale, ranging from 0 (“does not describe me well”) to 4 (“describes me very well”) and some items are scored in reverse fashion, resulting in a maximum score of 112.

### Statistical Analyses

We investigated internal consistency using Cronbach's alpha. To investigate the interrelatedness of the TriPM subscales as well as the association with external variables, we calculated Pearson's correlation coefficients. To lessen the risk of type I errors we set the significance level at 0.01 (two-tailed). We then further examined the association of the TriPM and other measures with multiple linear regression analyses. As running the regression models while controlling for age did not significantly affect the results, we did not include age as a co-variate in the final regression models. We used three independent variables in the models (Boldness, Meanness, and Disinhibition) and present the adjusted *R* square values. All models were evaluated according to standard principles and found to be acceptable regarding assumptions of linear regression. However, as discussed in for example, Lynam et al. (46) the high intercorrelations of Meanness and Disinhibition might result in problems of multicollinearity which limits the possibility of making reliable interpretations of the regression estimates, especially as partialling infers using residual scales rather than the original scales. Even though levels of tolerance and variance inflation factors were in the acceptable range, caution is warranted in the interpretation of the unique variance of particularly Meanness and Disinhibition in the models. All statistical analyses were conducted in SPSS (Version 26).

## Results

Internal consistency values, range, mean values, standard deviations, and correlation coefficients are reported in Table 1. Results of the regression models are presented in Table 2.

**Table 1:** Internal consistency values, range, mean values, standard deviations, and correlations matrix for all continuous variables.

**Variable**	**α**	**Range**	**Mean (SD)**	**Tri**	**Bold**	**Mea**	**Dis**	**PCL-R**	**F1**	**F2**	**F3**	**F4**	**N**	**E**	**O**	**A**	**C**	**IRI**	**BIS-11**	**Age**
Tri	0.96	32–165	85.9 (31.0)	–	**0.48**	**0.93**	**0.86**	**0.62**	0.14	**0.26**	**0.65**	**0.68**	−0.03	0.02	**−0.28**	**−0.80**	**−0.51**	**−0.65**	**0.73**	**−0.52**
Bold	0.82	7–54	33.7 (8.7)		–	**0.32**	0.09	**0.29**	**0.28**	0.16	**0.15**	**0.27**	**−0.51**	**0.34**	0.05	**−0.34**	0.17	**−0.35**	−0.00	−0.17
Mea	0.95	0–56	22.0 (15.1)			–	**0.74**	**0.54**	0.06	**0.30**	**0.58**	**0.60**	−0.02	−0.09	**−0.35**	**−0.79**	**−0.50**	**−0.72**	**0.69**	**−0.48**
Dis	0.93	0–59	30.2 (14.8)				–	**0.57**	0.07	0.15	**0.68**	**0.66**	**0.26**	−0.07	**−0.27**	**−0.67**	**−0.65**	**−0.43**	**0.82**	**−0.49**
PCL–R	0.86	1–39	20.7 (8.0)					–	**0.59**	**0.69**	**0.84**	**0.79**	−0.09	0.12	−0.15	**−0.50**	**−0.34**	**−0.45**	**0.46**	**−0.34**
F1	0.65	0–8	2.8 (2.0)						–	**0.43**	**0.29**	0.17	−0.19	0.15	0.09	**−0.20**	−0.07	−0.17	−0.02	0.03
F2	0.73	0–8	4.7 (2.2)							–	**0.45**	**0.35**	**−0.22**	0.06	−0.06	**−0.26**	−0.12	**−0.34**	0.07	−0.13
F3	0.73	0–10	5.6 (2.7)								–	**0.70**	0.06	0.07	**−0.20**	**−0.50**	**−0.46**	**−0.39**	**0.65**	**−0.49**
F4	0.81	0–10	5.2 (3.1)									–	0.01	0.07	**−0.22**	**−0.46**	**−0.31**	**−0.46**	**0.51**	**−0.42**
N	0.86	0–38	17.1 (8.9)										–	**−0.28**	0.03	−0.06	**−0.40**	**0.29**	**0.34**	−0.07
E	0.78	11–48	28.4 (7.1)											–	−0.01	0.12	**0.30**	0.10	−0.03	−0.03
O	0.57	5–37	21.6 (6.1)												–	**0.22**	0.11	**0.41**	**−0.40**	0.09
A	0.83	5–47	28.7 (8.5)													–	**0.56**	**0.58**	**−0.64**	**0.38**
C	0.87	9–47	31.2 (8.6)														–	**0.25**	**−0.70**	**0.34**
IRI	0.88	5–94	51.0 (17.8)															–	**−0.38**	**0.28**
BIS-11	0.93	35–106	71.0 (16.7)																–	**−0.46**
Age	–	22–65	37.1 (11.1)																	–

*Tri, TriPM Total Score (n = 191); Bold, Boldness (n = 191); Mea, Meanness (n = 191); Dis, Disinhibition (n = 191); PCL-R, Psychopathy Checklist—Revised (n = 191); F1, PCL-R Facet 1 (n = 191); F2, PCL-R Facet 2 (n = 191); F3, PCL-R Facet 3 (n = 191); F4, PCL-R Facet 4 (n = 191), N, NEO Neuroticism (n = 190); E, NEO Extroversion (n = 189); O, Openness (n = 189); A, Agreeableness (n = 189); C, Conscientiousness (n = 189); IRI, Interpersonal Reactivity Index (n = 186); BIS-11, Barratt Impulsiveness Scale (n = 191), Age, (n = 191). Correlations highlighted in bold are significant at the 0.01 level (2-tailed)*.

**Table 2:** Linear regression estimates of the associations between external variables and TriPM Boldness, Meanness and Disinhibition.

			***Bold***	***Mea***	***Dis***
**Linear model**	**F (df)**	***R* ^**2**^**	**β**	**β**	**β**
*PCL-R Total*	40.3 (3, 187)	0.38	**0.21**	0.14	***0.45***
*PCL-R Facet 1*	6.1 (3, 187)	0.07	***0.31***	−0.15	0.16
*PCL-R Facet 2*	7.0 (3, 187)	0.09	0.05	**0.38**	−0.14
*PCL-R Facet 3*	56.1 (3, 187)	0.47	0.05	0.15	***0.57***
*PCL-R Facet 4*	58.3 (3, 187)	0.48	**0.17**	0.17	***0.52***
*NEO Neuroticism*	36.0 (3, 186)	0.36	***−0.49***	−0.19	***0.44***
*NEO Extraversion*	12.1 (3, 185)	0.15	***0.43***	**−0.33**	0.13
*NEO Openness*	11.4 (3, 185)	0.14	0.19	***−0.43***	0.02
*NEO Agreeableness*	120.3 (3, 185)	0.66	**−0.13**	***−0.58***	***−0.23***
*NEO Conscientiousness*	59.8 (3, 185)	0.48	***0.29***	**−0.22**	***−0.51***
*IRI*	71.0 (3, 182)	0.53	−0.09	***−0.82***	0.18
*BIS-11*	151.7 (3, 187)	0.70	**−0.15**	***0.27***	***0.64***

*R^2^, Adjusted R squared. Bold, Boldness; Mea, Meanness; Dis, Disinhibition. Regression estimates with p <0.01 (2-tailed) are highlighted in bold, while estimates with p <0.001 (2-tailed) are highlighted in bold with italic*.

### Hypothesis 1. Interrelations of Boldness, Meanness, and Disinhibition

All domains were associated with TriPM total score (Table 1). Regarding the TriPM subscale intercorrelations, Meanness and Disinhibition proved to be the most strongly associated (*r* = 0.74, *p* < 0.001), followed by Meanness and Boldness (*r* = 0.32, *p* < 0.001). However, the correlation between Boldness and Disinhibition was weak and not significant (*r* = 0.09, *p* = 0.214).

### Hypothesis 2. The Convergence Between the TriPM and PCL-R

The TriPM total scores generally showed expected concurrency with PCL-R ratings and all TriPM domains correlated significantly to PCL-R total score (Table 1). Meanness and Disinhibition were strongly correlated with the Facets 3 (impulsive lifestyle; *r* = 0.58, *p* < 0.001 and *r* = 0.68, *p* < 0.001) and 4 (antisocial behavior; *r* = 0.60, *p* < 0.001 and *r* = 0.66, *p* < 0.001). Meanness also had a moderate correlation to Facet 2 (affective functioning; *r* = 0.30, *p* < 0.001). In contrast, the correlations of Boldness were mainly toward Facets 1 (interpersonal functioning; *r* = 0.28, *p* < 0.001) and 4 (antisocial behavior; *r* = 0.27, *p* < 0.001).

We then modeled the PCL-R total and facet scores using Boldness, Meanness, and Disinhibition as independent variables to account for TriPM domain co-variance, unanimously resulting in significant regression equations (Table 2). The results of the regression models showed that Boldness had a unique association to Facet 1 (β = 0.31, *p* < 0.001) and, to a lesser degree, to Facet 4 (β = 0.17, *p* = 0.004). Meanness was exclusively associated with Facet 2 (β = 0.38, *p* = 0.001). Lastly, Disinhibition was uniquely associated with Facet 3 (β = 0.57, *p* < 0.001) and Facet 4 (β = 0.52, *p* < 0.001), yet also demonstrated the strongest association with PCL-R total score (β = 0.45, *p* < 0.001).

### Hypothesis 3. Associations to Personality Traits Assessed by NEO-FFI, IRI, and BIS-11

The zero-order correlations between the TriPM domains and general personality dimensions demonstrated specific FFM trait profiles for the different subscales. Meanness was negatively correlated to Agreeableness (*r* = −0.79, *p* < 0.001), Conscientiousness (*r* = −0.50, *p* < 0.001) and Openness (*r* = −0.35, *p* < 0.001). Disinhibition also presented negative correlations to Agreeableness (*r* = −0.67, *p* < 0.001), Conscientiousness (*r* = −0.65, *p* < 0.001), and Openness (*r* = −0.27, *p* < 0.001), yet had a correlation to Neuroticism (*r* = 0.26, *p* < 0.001). Boldness, on the other hand, performed differently and demonstrated in particular low Neuroticism (*r* = −0.51, *p* < 0.001) and high Extraversion (*r* = 0.34, *p* < 0.001), although it also exhibited low Agreeableness (*r* = −0.34, *p* < 0.001). In regards to empathy, all TriPM domains were significantly correlated to low IRI scores, although for Meanness this was particularly strong (*r* = −0.72, *p* < 0.001). Lastly, both Meanness and Disinhibition displayed a strong correlation to impulsivity measured with BIS-11 (*r* = 0.69, *p* < 0.001 and *r* = 0.82, *p* < 0.001).

Our regression models supported that, even after removing the shared variance between them, the TriPM domains demonstrated unique associations to the FFM personality traits (Table 2). Boldness was the only triarchic domain associated with high Extraversion (β = 0.43, *p* < 0.001) and Conscientiousness (β = 0.29, *p* < 0.001) as well as low Neuroticism (β = −0.49, *p* < 0.001). Meanness in turn had an exclusive association with low scores on Openness (β = −0.43, *p* < 0.001) and IRI (β = −0.82, *p* < 0.001). Lastly, Disinhibition was uniquely associated with elevated scores on Neuroticism (β = 0.44, *p* < 0.001). Even though BIS-11 exhibited positive associations with both Meanness and Disinhibition, it was particularly for the latter (β = 0.27, *p* < 0.001 and β = 0.64, *p* < 0.001, respectively).

## Discussion

The aim of the current study was to examine the psychometric properties of the TriPM. Specifically, we investigated if the Swedish translation showed expected subscale intercorrelations and associations with expert-rated psychopathy, general personality dimensions, empathy, and impulsivity.

Our first hypothesis stated that in regards to TriPM domain scores, Meanness and Disinhibition would be strongly associated, while Boldness would mainly be associated with Meanness and only demonstrate a small association to Disinhibition. This hypothesis was partly confirmed, as Meanness and Disinhibition indeed proved to be the most strongly associated, followed by Meanness and Boldness. However, even though all domains were associated with TriPM total score, Boldness and Disinhibition did not correlate significantly in our study.

When the triarchic model was developed, Patrick et al. (3) emphasized the importance of treating psychopathy as a combination of the separate domains rather than as an overarching construct. Although the model suggests that the triarchic domains partially overlap due to their shared biological etiology, they should also be adequately differentiated from each other, that is boldness, meanness and disinhibition should be measured as clearly defined constructs. Despite the fact that research utilizing the TriPM is steadily growing, there are few prior studies that have presented interrelatedness of the triarchic constructs in forensic samples, where prototypic psychopathy is more readily found (18, 20, 21, 24, 35, 47, 48). Our results are however in line with these, indicating that the instrument is utilizable in a Swedish forensic setting.

One important avenue in assessing the psychometric evidence of the TriPM is to investigate if the factor structure of the scale conforms to the theoretical model. So far, reports shedding light on the measurement model of the TriPM, such as using factor analysis, are sparse. The studies that are available do not provide clear evidence for the three dimensions in the original measurement model, based on standard criteria of good model fit (8, 49–51). Furthermore, the items of the TriPM display varying associations to the hypothesized factors, with several items loading weakly to the intended factor, as well as cross-loading to the other factors (8, 50). Additionally, in line with the remarkably high correlation of TriPM Disinhibition and Meanness in our sample, Sleep et al. (13) demonstrated that the Meanness and Disinhibition scales show limited discriminant validity, possibly due to their substantial intercorrelations and inclusion of explicit antisocial items. As a way to address this, Shou et al. (52) have suggested deleting some items from the Meanness and Disinhibition subscales to improve their separability. They also found evidence pointing to a multi-dimensional model of Boldness. In addition, the lack of association between the TriPM Disinhibition and Boldness may put to question if all domains are meaningfully related within the same construct. Roy et al. (51, 53), by performing an item-level factor structure analysis of the TriPM, found the current three domains to be inherently multidimensional and instead proposed a 7-factor structure based of the psychometric instrument. They suggest that the psychometric weaknesses of the instrument, such as lack of internal construct validity, risk leading to statistical washout effects and a theoretical over-simplification of the psychopathic personality. Patrick et al. (54), however, argue that the TriPM should be viewed as an item-based factor scale which is developed to encompass the broad factors from its parent multiscale instruments (i.e., ESI and BI) as well as utilizing external validity as evidence of the measurement model.

In order to explore the factor structure of the TriPM in the current study population, we performed a confirmatory factor analysis as previously described by Roy et al. (51) with both a three-factor model in order to evaluate the triarchic constructs, and separate analyses for each TriPM scale to assess if they were unidimensional. The analysis is available in the Supplementary Table 1. Neither the three-factor model nor the individual single-factor models indicated adequate model fit in regards to criteria proposed by Hu and Bentler (55). We interpret these results with caution, considering our small population size both in absolute numbers and in regards to the observed variables, yet they are in line with previous research. The critique of the psychometric properties of the TriPM may, at least in part, explain that our results were somewhat divergent from the theoretical model.

Our second hypothesis predicted that psychopathic traits assessed by TriPM would be convergent with PCL-R based psychopathy and we expected to find associations between Boldness and Facet 1 and Facet 2, Meanness and Facet 2, as well as Disinhibition and Facet 3 and 4. The TriPM domains and PCL-R facets were mainly associated in the anticipated manner. The exception to this was Boldness which, when controlling for variance shared with the other domains, was not associated to affective functioning as hypothesized. Unexpectedly, we instead found that it was linked to antisocial behavior. In line with this, all TriPM domains in our sample correlated significantly with PCL-R total score (as opposed to previous studies where this was not found for the TriPM Boldness scale). These findings in regards to TriPM Boldness can potentially be explained by the fact that being more prone to risk-taking, fearlessness and carefreeness may predispose for antisocial behavior. This would mean that boldness, at least in offender samples, might not be an adaptive trait. Previous studies using the PCL (18, 20) have reported similar results, although Sellbom and colleagues used the Psychopathy Checklist Screening Version (PCL:SV) and therefore did not report the antisocial facet due to low internal consistency in their sample. However, the observation of Wall et al. (18) that TriPM Boldness did not present incremental validity for antisocial behavior (Facet 4) beyond the variance shared with ASPD, suggests that the association between scores on Boldness and Facet 4 might be explained by the high occurrence of ASPD in our sample.

Our third hypothesis aimed at translating the domains of triarchic psychopathy in terms of general personality dimensions and other psychopathy-essential traits such as empathy and impulsivity. The results supported that TriPM Boldness could be described in terms of high FFM extraversion and low neuroticism (i.e., resilience). However, in the current sample, it was also to some extent characterized by low agreeableness (i.e., antagonism), even when accounting for the variance explained by Meanness. Again, this suggests that boldness might not be an adaptive feature in the current sample.

In regards to TriPM Meanness and Disinhibition, both subscales performed similarly with negative associations to the general personality domains of agreeableness (i.e., self-centered in motivation and behavior) and conscientiousness (i.e., lack of direction), as well as positively associated with impulsivity. These findings were expected when considering the subscale overlap of the triarchic model of psychopathy. The strength of the associations between the triarchic domains and personality dimensions were also in line with expected patterns, as we found a strong correlation between meanness and low agreeableness, as well as disinhibition and impulsivity. Additionally, the unique association between TriPM Disinhibition scale and high neuroticism (i.e., emotional instability) was anticipated. However, specific to the Meanness scale was associations with low openness and extraversion. This is not clearly implied by the theoretical idea of the construct, but an association with low openness was also reported in the only previous study investigating TriPM and FFM in offenders (24). This indicates that in prison inmates, TriPM Meanness may be associated with being conservative in your thinking. This finding does not seem be exclusive for forensic populations, as this outcome has also been reported in a community sample (33). In regards to the association between the Meanness scale and low extraversion, this implies a tendency of being reserved and aloof, which has also previously been noticed (27, 28, 30), although it is not a consistent observation.

To explore these findings, we can view them from the theoretical assumptions of the triarchic model. The theory postulates that the domains of boldness, meanness and disinhibition stem from two etiological pathways, difficult temperament and low fear (3). Meanness and disinhibition thus have the suppositional common etiology of difficult temperament, which could explain the intertwined associations regarding impulsivity, conscientiousness, and agreeableness. This seem to have a basis of both genetic and environmental underpinnings, demonstrated in a twin-study by Tuvblad et al. (56). They found that the intercorrelation between the TriPM Meanness and Disinhibition scales was partly explained by shared genes (26%), while shared and non-shared environmental factors explained the remaining overlap.

Still, the divergent associations of the TriPM Meanness and Disinhibition scales outlines meaningful differences. TriPM Meanness was highly associated with a lack of empathy, and thus is closely related to one of the key features of psychopathy. However, against expectation, TriPM Disinhibition demonstrated a small positive association, although not significant, to empathy as measured with IRI. This is problematic in regards to the construct of psychopathy and similar findings have been presented previously (24, 38–40), yet contradicting results have also been reported (41). A higher expression of TriPM Disinhibition thus indicated a greater degree of empathy as well as neuroticism. The latter is a feature not paired with psychopathy in general, but which is often discussed in terms of secondary psychopathy [e.g., (57)]. A potential explanation may stem from the overlap between TriPM Disinhibition and ADHD symptoms, which has previously been discussed in regards to the current sample of prison inmates (37). A previous study by Retz et al. (58) found that ADHD and psychopathy assessed with PCL:SV among offenders were clearly separable constructs, though they intersect in regards to the items that measure impulsive behavior in their respective diagnostic instruments. While subjects with ADHD may be more prone to exhibit antisocial behavior, they are however not susceptible to development of the interpersonal or affective traits of psychopathy (36). The high prevalence of self-reported ADHD in the current population, constituting of approximately a third of participants, may partially explain the positive associations between TriPM Disinhibition, neuroticism and empathy, as a constructional overlap between psychopathy and ADHD consequently might “muddle” the results of the disinhibition domain. However, as these diagnoses was not confirmed through a review of the subject's medical files, there is the possibility that some subjects either over- or underreported said diagnosis. Even though our frequency of ADHD may seem high, it is in line with previous reported meta-analytic prevalence estimates in incarcerated adult populations (26%) (59) as well as evaluated prevalence when investigating Swedish long-term prison inmates (40%) (60).

Furthermore, according to the underlying assumptions of the triarchic model, boldness and disinhibition do not share the same etiological contributors (i.e., the complex mechanisms of genetic constitution, environmental influences, and their respective interactions on each other). While disinhibition is thought to stem from a lack of emotional regulation and behavioral restraints manifesting in a difficult temperament, boldness is instead theoretically related to a biological disposition of low fear leading to a reduced sensitivity to stressful stimuli as well as a proneness to reward-seeking instead of aversion to punishment. The assumption of these differing pathogenic mechanisms gains support from the divergent association patterns of TriPM Boldness and Disinhibition, with the exception of low agreeableness which was linked to all TriPM domains to varying degrees. On the other hand, meanness is thought to share its basis in both of these etiological pathways and therefore should be related to both boldness and disinhibition. In the current study, the TriPM Boldness and Meanness scales revealed no meaningful similarities in associations and thus our results do not support this assumption. Possibly, this can be partly explained by the emphasis put on externalizing and antisocial behavior in the TriPM operationalization, which might accentuate the similarities of the Meanness and Disinhibition scales, while masking the interrelatedness of Meanness and Boldness. As an alternative interpretation, our results might also point to the construct of boldness lacking importance for psychopathy (9, 13, 31), especially in a forensic context. Nevertheless, while TriPM Meanness was not associated with PCL-R total score, Boldness was. As the PCL-R to date is regarded as the most accepted measure of psychopathy, this association suggests that boldness is of relevance as part of the psychopathic construct.

Our results found that the Swedish translation of the TriPM is an effective method to assess psychopathic traits in a forensic context, where these traits are more commonly prevalent. Even though it doesn't replace expert assessments as the most reliable diagnostic instrument for psychopathy, we believe that it has an application in both clinical settings as well as research, primarily as an easily administered tool to screen for psychopathic traits. In prison services as well as in forensic psychiatry, identifying inmates with a high risk of behavioral disturbances and misconduct at an early stage offer the ability to focus investigative resources and take this into account when making treatment plans. In a study of 871 psychiatric patients, Skeem et al. (57) found that treatments aiming to reduce the risk of violence were effective for psychopathic participants, even though they seemed to benefit from it being more intense and extensive than for participants without the traits. In regards to the application of the TriPM in the field of research, the instrument additionally tenders the ability to quantify different aspects of the psychopathic personality construct. This is of importance in trying to understand the multifactorial and possibly distinct biological processes underlying psychopathy. Additionally, it is useful in evaluations of potential treatments, as the psychopathology of the disorder is complex and leads to a heterogeneity in the expression of the personality and behavior among individuals with psychopathic traits. The current study contributes to evidence for the TriPM being used in its Swedish translation and thus for potential applications in both clinical practice and furthering the research of psychopathy.

The TriPM operationalizes the triarchic model of psychopathy by using a self-report, which could be considered complicated especially when dealing with a population prone to antisocial behaviors such as carefree lying, conning and manipulation. A concern is that respondents try to present themselves more favorably, alternatively that they are susceptible to self-deception and more inclined to an overtly positive self-image. This was addressed in a meta-analysis by Hildebrand et al. (61) examining 19 studies assessing dynamic risk factors in self-report measures, which found that socially desirable responding did not compromise their effectiveness in forensic settings, even though antisocial personality traits was a potential problematic trait of concern. The other main concern in addition to misleading self-presentation when utilizing self-report questionnaires is inattentive responding, found to be prevalent in ~10% of subjects in community populations (62). One could argue that respondents in forensic populations in general have a higher degree of attention deficits and lower educational level, and consequently are more likely to be easily distracted or interpret items incorrectly. The TriPM does not have a validity scale incorporated and thus the responses from subjects that are unconcerned or unmotivated can severely impede the interpretation of the instrument. As a mean to address this, we utilized TAPIR, a procedure developed by Mowle et al. (42) to identify erratic responding by examining the responses on 13 highly-correlated item-pairs and translating this to a dimensional scale. The method was developed in both community and forensic populations, and have suggested cut-off values for both groups, respectively. The TAPIR has been examined in several languages, including Swedish, and has been found to offer cross-language generalizability (43). In the current study, <2% of participants were identified as inconsistent responders, which is generally lower than expected based on community populations. This may be due to the fact that the self-report instrument was administered individually as part of the study protocol and thus may have heightened the attention and motivation for completion among participants. This may not be representative for participation when employed in a clinical context and warrants further examination.

A possible caveat in utilizing the triarchic model to assess psychopathy, may be that it is under-inclusive of some important psychopathic traits, at least as measured by the TriPM. From a clinical view-point, grandiosity, sense of entitlement and arrogance are highly relevant personality traits that often severely affect social interactions and lead to self-centered behavior, and thus is of importance in assessing the disorder. In comparison to other conceptualizations of psychopathy such as the Comprehensive Assessment of Psychopathic Personality (CAPP) (63) and the PCL-R model, the triarchic model does not seem to emphasize the narcissistic aspects of psychopathic personality. Specifically, using the CAPP model, a prototypically psychopathic person would among other traits be described as domineering, deceitful and manipulative, as well as self-centered, self-aggrandizing and self-justifying. Donnellan and Burt (29) argue that narcissistic traits intersect with the TriPM domains, with grandiosity being correlated to the Boldness domain, while the more socially problematic feature of entitlement is linked to Meanness. In our study, we did not specifically study the links between narcissism and the triarchic model. However, in regards to the PCL-R interpersonal and affective facets that are thought to encompass narcissistic expressions in the PCL-R model, we found they were linked to TriPM scores through Boldness and Meanness, respectively. Even though the domains of the triarchic model overlap narcissism, at least in part, the items of the TriPM do not seem to directly pinpoint these symptom and further examinations of the constructs' interconnections are warranted.

### Limitations

Even though we consider the use of PCL-R as a strength of this study, for practical reasons we did not have access to independent raters and thus could not investigate inter-rater reliability estimates. The sample consisted of incarcerated offenders, which is a group that is previously understudied regarding the TriPM, but the use of a comparison group would have been of value. A general disposition of externalizing behavior such as early behavior problems, drug abuse, aggressiveness, and ADHD symptoms are potential confounding factors and weakens the possibilities of generalizing the results to other populations. Furthermore, as all participants were men, the results are not generalizable to women. In addition, on account of the study being drawn from a genetic study, all participants were of Swedish ethnicity, which also limits the findings. Unfortunately, as the current study used the short version of NEO to measure personality traits according to the FFM, we did not have access to the domain facets, which could have given more depth to the discussion of the underlying traits of the triarchic dimensions, especially in entangling the interrelation and distinction between the TriPM Meanness and Disinhibition scales. That would also have permitted us to investigate the nomological network of the TriPM from a FFM perspective, particularly examining the incremental value of the TriPM compared to a FFM based profile of the triarchic model as discussed by Hyatt et al. (34).

## Conclusion

The current study provides further evidence for the triarchic model of psychopathy, contributing to amend the lack of studies using the TriPM in a forensic context. Overall, our results were generally in line with previous research and the theoretical descriptions of the triarchic model. Even though the sample was limited to male offenders, all variables of interest showed a large variance giving us the opportunity to study the traits of interest in their full dimensionality. Granted that the TriPM has some psychometric issues that ought to be addressed, most notably regarding the impact of antisocial behavior, the results of the current study generally show expected associations to other relevant constructs as well as clear and expected differentiations of the patterns of association to the respective domain, in support of the theoretical framework of the model. Furthermore, the results are in line with previous studies, which adds further to the validity evidence for using the Swedish translation of the TriPM in a novel context, in this case a Nordic country.

## Data Availability Statement

The raw data supporting the conclusions of this article will be made available by the authors, without undue reservation.

## Ethics Statement

The studies involving human participants were reviewed and approved by Regional Ethical Review Board of Stockholm (#2014/1192-31/1). The patients/participants provided their written informed consent to participate in this study.

## Author Contributions

MP: study design, hypothesis formulation, data acquisition, statistical analysis, interpretation of data, manuscript writing, and final approval of the version to be submitted. HÖ: hypothesis formulation, statistical analysis, interpretation of data, manuscript writing, and submitting and corresponding author. JL: study design, hypothesis formulation, data acquisition, interpretation of data, and final approval of the version to be submitted. PG: study design, statistical analysis, interpretation of data, and final approval of the version to be submitted. KH: conceived the study, study design, hypothesis formulation, statistical analysis, interpretation of data, revised the manuscript, and final approval of the version to be submitted. All authors contributed to the article and approved the submitted version.

## Funding

This research was supported by the Swedish National Board of Forensic Medicine and funded in part by a scholarship from the Swedish Neuropsychological Society as well as a grant from the Bror Gadelius trust fund.

## Conflict of Interest

The authors declare that the research was conducted in the absence of any commercial or financial relationships that could be construed as a potential conflict of interest.

## Publisher's Note

All claims expressed in this article are solely those of the authors and do not necessarily represent those of their affiliated organizations, or those of the publisher, the editors and the reviewers. Any product that may be evaluated in this article, or claim that may be made by its manufacturer, is not guaranteed or endorsed by the publisher.

## Footnotes

^1^The effect of excluding the subjects based on TAPIR score did not significantly alter the results, as changes in zero-order correlation coefficients were small (changes in *r* ranging from −0.019 to 0.021, *M* = −0.001).
