# Supplementary material for: Investigating the Validity Evidence of the Swedish TriPM in High Security Prisoners Using the PCL-R and NEO-FFI
Source: Front Psychiatry. 2021 Nov 19;12:704516. doi: 10.3389/fpsyt.2021.704516 (PMC8640174; doi:10.3389/fpsyt.2021.704516)
Supplement: Supplementary file 1 [file Data_Sheet_1.docx]

Investigation the Validity Evidence of the Swedish TriPM in High Security Prisoners using the PCL-R and NEO-FFI

Malin Pauli^1,2†^, Hannibal Ölund Alonso^1,3†*^, Jenny Liljeberg^1,2^, Petter Gustavsson^1^, Katarina Howner^1,2^

^1^ Department of Clinical Neuroscience, Karolinska Institutet, Stockholm, Sweden

^2^ National Board of Forensic Medicine, Stockholm, Sweden

^3^ Forensic Psychiatric Care Stockholm, Stockholm Healthcare Services, Stockholm, Sweden

^†^These authors have contributed equally to this work and share first authorship

*** Correspondence:**Hannibal Ölund Alonso
hannibal.olund.alonso@ki.se

Supplementary Material

# Factor analysis

The triarchic structure of the instrument was tested using a three-factor model with items assigned to corresponding TriPM scale of Boldness, Meanness, or Disinhibition and with the factors allowed to freely correlate. Separately, single-factor models were used for the items on each TriPM scale in order to assess if could were unidimensional and thus could be explained by a dominant factor. Statistical analyses were performed in R (R Core Team, 2020) utilizing the lavaan package (Rosseel, 2012).

**Table 1.** Goodness-of-fit statistics for robust weighted least squares mean and variance adjusted confirmatory factor analysis models of the Triarchic Psychopathy Measure.

| TriPM model | χ^2^ (df) | RMSEA | 90 % CI | SRMR | CFI | TLI |
| --- | --- | --- | --- | --- | --- | --- |
| Three-factor model | 2544.67 (1592)* | .06 | .06, .06 | .10 | .82 | .81 |
| *Single-factor models* |  |  |  |  |  |  |
| Boldness scale (19 items) | 389.46 (152)* | .09 | .08, .10 | .11 | .64 | .59 |
| Meanness scale (19 items) | 401.31 (152)* | .09 | .08, .10 | .07 | .88 | .86 |
| Disinhibition scale (20 items) | 400.80 (170)* | .09 | .07, .10 | .07 | .88 | .86 |
| *Note*. *TriPM* Triarchic Psychopathy Measure; *RMSEA* root mean square error of approximation; *CI* confidence interval; *SRMR* standardized root mean square residual; *CFI* comparative fit index; *TLI* Tucker-Lewis Index. * p < 0.001. | | | | | | |

# References

R Core Team (2020). *R: A language and environment for statistical computing.* Vienna, Austria: R Foundation for Statistical Computing Available at: https://www.r-project.org/.

Rosseel, Y. (2012). Lavaan: An R package for structural equation modeling. *J. Stat. Softw.* doi:10.18637/jss.v048.i02.
